# Maximal bite force, facial morphology and sucking habits in young children with functional posterior crossbite

**DOI:** 10.1590/S1678-77572010000200008

**Published:** 2010

**Authors:** Paula Midori CASTELO, Maria Beatriz Duarte GAVIÃO, Luciano José PEREIRA, Leonardo Rigoldi BONJARDIM

**Affiliations:** 1 DDS, PhD, Department of Biological Sciences, Federal University of São Paulo, Diadema, SP, Brazil.; 2 DDS, PhD, Department of Pediatric Dentistry, Piracicaba Dental School, State University of Campinas, Piracicaba, SP, Brazil.; 3 DDS, PhD, Department of Physiology, Federal University of Lavras, Lavras, MG, Brazil.; 4 DDS, PhD, Department of Physiology, Dental School of the Federal University of Sergipe, Aracaju, SE, Brazil.

**Keywords:** Bite force, Face, Sucking behavior, Malocclusion, Pacifiers

## Abstract

**Objective:**

The maintenance of normal conditions of the masticatory function is determinant for the correct growth and development of its structures. Thus, the aims of this study were to evaluate the influence of sucking habits on the presence of crossbite and its relationship with maximal bite force, facial morphology and body variables in 67 children of both genders (3.5-7 years) with primary or early mixed dentition.

**Material and methods:**

The children were divided in four groups: primary-normocclusion (PN, n=19), primary-crossbite (PC, n=19), mixed-normocclusion (MN, n=13), and mixed-crossbite (MC, n=16). Bite force was measured with a pressurized tube, and facial morphology was determined by standardized frontal photographs: AFH (anterior face height) and BFW (bizygomatic facial width).

**Results:**

It was observed that MC group showed lower bite force than MN, and AFH/ BFW was significantly smaller in PN than PC (t-test). Weight and height were only significantly correlated with bite force in PC group (Pearson’s correlation test). In the primary dentition, AFH/BFW and breast-feeding (at least six months) were positive and negatively associated with crossbite, respectively (multiple logistic regression). In the mixed dentition, breastfeeding and bite force showed negative associations with crossbite (univariate regression), while nonnutritive sucking (up to 3 years) associated significantly with crossbite in all groups (multiple logistic regression).

**Conclusions:**

In the studied sample, sucking habits played an important role in the etiology of crossbite, which was associated with lower bite force and long-face tendency.

## INTRODUCTION

Breast-feeding encourages normal growth and development of the alveolar processes and stomatognathic structures, correct intermaxillary relationship and nose breathing^20^. If “suck need” is not satisfied during regular feeding, it may be fulfilled by a sucking habit. Some studies have reported the effects of persistent nonnutritive sucking on sagittal and vertical dimensions of the maxilla and the mandible, dependent on the intensity and the duration of the habit^12,18^. Posterior crossbite occurs frequently in children, as a result of genetic or environmental influences (for example, nonnutritive sucking habits and mouth breathing), or a combination of both, and has been associated with asymmetrical growth and function of the hard structures and muscles^1,6,26,29^. Betts, et al.^2^ (1995) stated that a posterior crossbite does not confine itself to dental displasias but is more often related to an underlying skeletal problem.

Bite force is one of the components of the chewing system, which may be influenced by dental occlusion, craniofacial morphology and masticatory muscle thickness. Its magnitude increases with age, with teeth in occlusal contact, and with increasing number of erupted teeth^26^. Craniofacial morphology evaluation is also an important tool in clinical practice and research, and can be achieved by different approaches, including photographic analyses, which is an inexpensive method, does not expose the patient to unnecessary irradiation, and can provide the evaluation of external craniofacial structures^7,27^.

In this way, the purposes of this study were to evaluate the association of sucking habits with the presence of posterior crossbite among children in the primary and early mixed dentition, and its relationship with maximal bite force and facial dimensions.

## MATERIALS AND METHODS

This cross-sectional study comprised a convenience sample formed by healthy children of both genders aged from 3.5 to 7 years, who were to start treatment in the Department of Pediatric Dentistry and from day care centers. All children and their parents consented to participate in the study, which was approved by the Ethics Committee of our institution (protocol nos. 147/2001 and 148/ 2002). They were selected after a complete anamneses and clinical examination, when body weight and height, morphological occlusion, stage of the dentition (primary/early mixed dentition), and the presence of normocclusion or unilateral posterior crossbite (functional, involving canine and primary molars) were verified^25^. The inclusion criterion for crossbite was the presence of mild bilateral constriction of the upper arch and a mandible shifting due to the presence of tooth interference. Children with structure/number of teeth alterations and oral tissue and severe obstruction of upper airways were excluded. A total of 67 subjects were selected and distributed in four groups: PN - primary-normocclusion, PC - primary -crossbite, MN – mixed-normocclusion, and MC – mixed-crossbite (Table 1). The exclusion criteria for normocclusion groups were the presence of signs and/or symptoms of temporomandibular dysfunction^4^, and previous orthodontic treatment.

**Table 1:** Mean (SD) values for age, body variables, facial morphology and maximal bite force (BF) for all groups and the results of statistical analysis

**Group**	**PC**	**PN**	**Univariate logistic regression**	**Multivariate logistic regression**	**MC**	**MN**	**Univariate logistic regression**	**Multivariate logistic regression**
**n**	**19**	**19**			**16**	**13**	***p* -value**	***p* -value**
	**Mean (SD)**	**Mean (SD)**	***p* -value**	***p* -value**	**Mean (SD)**	**Mean (SD)**		
Gender	9Fand 10M	5F and14M	-	-	11F and 5M	6F and 7M	-	-
Age (months)	59.47	58.42	NS	NA	73.25	72.69	NS	NA
	(7.21)	(8.50)			(7.28)	(6.17)		
Weight (Kg)	19.34 ‡	19.79	NS	NA	23.31	25.72	NS	NA
	(2.25)	(4.17)			(5.81)	(4.65)		
Height (m)	1.10 ‡	1.09	NS	NA	1.18	1.18	NS	NA
	(0.06)	(0.07)			(0.07)	(0.05)		
AFH/BFW	0.78 *	0.75 *	0.038	0.016	0.78	0.75	NS	NA
	(0.03)	(0.03)			(0.05)	(0.03)		
BF (N)	277.75	280.46	NS	NA	316.42 †	352.81 †	0.045	NS
	(53.27)	(48.31)			(52.16)	(23.67)		

PC, primary dentition-crossbite; PN, primary dentition-normal occlusion; MC, mixed dentition-crossbite; MN, mixed dentition-normal occlusion; AFH, anterior facial height; BFW, bizygomatic facial width.

* *p* <0.05 unpaired f-test for AFH/BFW comparison between primary dentition groups.

† *p* <0.05 unpaired f-test for BF comparison between mixed dentition groups.

‡ *p* <0.05 Pearson correlation test between BF and body variables.

NA, not applicable; NS, not significant; SD, standard deviation; F, female; M, male

Data regarding the history, presence and duration of sucking habits were obtained from the parents/guardians, considering the following parameters: - breast-feeding over a period of at least six months (exclusive or not exclusive); bottle-feeding for 1 year or more; - nonnutritive sucking habit (pacifier or thumb sucking) that persisted up to the age of 3 years.

All analyses were done by the first author (PMC).

### Maximal bite force measurement

Maximal bite force was assessed with a pressurized transducer tube constructed with a flexible material (10 mm diameter), and connected to a sensor element (MPX5700 Motorola, Austin, TX, USA). The tube was placed bilaterally over the primary molars, and the recordings were performed three times, with an interval of two minutes. The children were seated in an upright position with the head in natural posture and they were instructed to bite the tube as forcefully as possible, and the final value was determined as the average of the three measurements (accuracy of 0.1 N). The measurements were transferred to a computer in pounds per square inch (PSI) and later converted into Newtons (N).

### Facial morphometry by photographic evaluation

**Figure 1:**
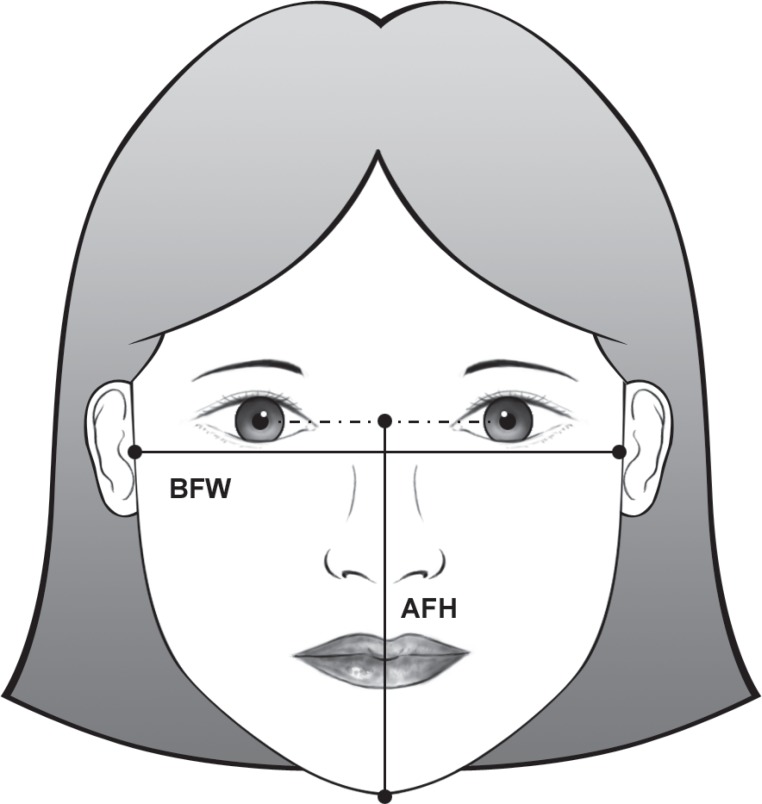
Facial dimensions: AFH, anterior face height (the linear distance between the interpupillary plane and the inferior margin of the menton); BFW, bizygomatic facial width (the linear distance between the bilateral most exterior points of the zygomatic arches)

Facial dimensions were determined by measuring standardized frontal photographs (10x15 cm), taken from a digital camera and automatic flash (Canon EOS Digital DS6041, 6.3MP, Canon Inc., Ohta-ku, Tokyo, Japan), fixed on a tripod. The children remained in the standing position in front of a white background, under a natural light and in relaxed position, with about 20 cm of legs distance in order to give stability. The head was positioned with the saggital plane perpendicular and Frankfort plane parallel to the horizontal plane. The dimensions^3,7^ were hand traced on acetate paper and measured using digital caliper accurate to 0.01 mm and are detailed in Figure 1. Dimensions ratio and printed photographs were used to reduce errors.

### Measurement errors

The reliability of the measurements for bite force and facial dimensions ratio was determined in 15 randomly selected children not included in this study. Two repeated measurements (x_1_, x_2_), at interval of 15 days, were taken and the differences between the two sets of measurements were calculated by Dahlberg’s formula: Method Error (ME) = √Σ (m_1_- m_2_)^2^/2n. The error of the method for maximal bite force and facial dimensions ratio were 16.28 N and <0.01, respectively.

### Statistics

Logistic regression models with the binary endpoint of crossbite (yes, no) were fit to evaluate the association between the presence of crossbite as the dependent variable and the following independent variables: bite force, AFH/BFW (anterior face height/bizygomatic facial width), and nutritive and nonnutritive sucking habits, controlling for age, weight and height. First, univariate models identified a set of variables that were independently associated with the presence of crossbite in each stage of dentition. Following, the variables that were significantly associated (p<0.05) were taken as potential predictors of crossbite and were used as covariates in the multivariate logistic regression analysis.

The correlation between bite force and age, weight, height, and AFH/BFW were estimated for the groups using Pearson correlation coefficient. Fisher’s exact test was applied in order to verify the differences in proportions of children with crossbite and normocclusion, considering the nutritive and nonnutritive sucking habits. All calculated p values were two-sided, and values less than 0.05 were considered statistically significant. The statistic analysis was performed using Intercooled Stata 7.0 (STATA Corporation, College Station, TX, USA).

## RESULTS

Tables 1 and 2 show the sample distribution according occlusion and stage of dentition, the information concerning age, body variables, facial dimensions and bite force, and the descriptive statistics. The MC group presented bite force values significantly smaller than group MN, whereas in the primary dentition, AFH/BFW ratio was significantly smaller in PN group (p<0.05). Body variables were only significant correlated with bite force in PC group.

**Table 2:** Sample distribution according to the presence of nutritive and nonnutritive sucking habits and the results of statistical analysis

				**Logistic regression**					**Logistic regression**	
**Group**	**PC**	**PN**	**Fisher's**	**Univariate analysis**	**Multivariate analysis**	**MC**	**MN**	**Fisher's**	**Univariate**	**Multivariate**
**n**	**19**	**19**	**Exact test**			**16**	**13**	**Exact test**	**analysis**	**analysis**
	**n %**	**n %**	***p* -value**	***p* -value**	***p* -value**	**n %**	**n %**	***p* -value**	***p* -value**	***p* -value**
Nonnutritive	10 (52.6%)	3 (15.8%)	0.022	0.022	0.049	13 (81.3%)	3 (23.1%)	0.003	0.004	0.020
sucking										
Breast	7 (36.8%)	15 (79.0%)	0.007	0.012	0.040	4 (25.0%)	10 (76.9%)	0.009	0.009	NS
feeding										
Bottle-	18 (94.7%)	16 (84.2%)	NA	NA	NA	16 (100.0%)	12 (92.3%)	NA	NA	NA
feeding										

PC, primary dentition-crossbite; PN, primary dentition-normal occlusion; MC, mixed dentition-crossbite; MN, mixed dentition-normal occlusion.

NA, not applicable; NS, not significant.

According to the multiple logistic regressions, AFH/BFW ratio, nonnutritive sucking habits and breast-feeding were the major independent predictors of crossbite in primary dentition (p<0.05). In the mixed dentition, univariate analyses showed that children with lower bite force and the absence of breast-feeding were significantly more likely to have a posterior crossbite; but they can not be considered predictors of this malocclusion, due to the no significant levels reached in the multiple logistic models. Multivariable analyses showed that nonnutritive sucking habits were significantly associated with the presence of crossbite in the mixed groups, that is, a nonnutrive sucking habit can predict the development of this malocclusion in both evaluated dentitions. Fisher’s exact test also showed significant association between sucking habits and crossbite in both stages of the dentition. Bottle-feeding for 1 year or more was highly prevalent in both groups of the mixed dentition; for this reason, this variable was removed from the models.

## DISCUSSION

Possible etiologies of crossbite may include prolonged retention or premature loss of primary teeth, crowding, palatal cleft, genetic influence, arch deficiencies, abnormalities in tooth anatomy or eruption sequence, non-nutritive sucking habits, oral respiration during critical growth periods, and temporomandibular disorders^8,20^. Since an untreated crossbite is thought to be detrimental for function^6,20,22^,the early diagnosis and functional examination must be considered in clinical practice. Past studies^12,19^ emphasized the importance of unfavorable factors on the growth and development of the oral and facial structures, as well as the influence of favorable factors that places beneficial orthopedic forces on the jaws, such breast-feeding, and the need for attention to the magnitude of malocclusion in childhood. A reduced electromyographic activity for the masseter muscle in bottle-fed babies may be observed when compared with that breast-fed^10^. According to the results found, the absence of breast-feeding showed to be a potential predictor for the development of crossbite in the primary stage, although the use of bottle-feeding for 1 year or more has shown to be highly prevalent in the studied sample. Larsson^18^ (2001) observed the development of interfering contacts in primary canines and midline shift among pacifier- and digitsuckers; in these cases, the author concluded that parents should be instructed to reduce the “in the mouth time” of the habit. This effect occurs because when the teat of the pacifier is kept in the mouth, the tongue will be forced to a lower position in the anterior part of the mouth, thereby reducing the palatal support of the upper primary canines and molars against the pressure of the cheeks, resulting in a narrower upper arch. According to Katz, Rosenblatt and Gondim^12^ (2004), the importance of genetic factors in the etiology of malocclusions seems to be less than environmental factors.

Determination of bite force magnitude has been widely used in studies^5,23^ to understand mastication mechanisms and its relationship with stomatognathic structures. In agreement with previous studies^6,26^, the studied sample showed significant difference in the maximum bite force between children with and without crossbite in the mixed dentition. This find may be due to alterations in certain masticatory parameters, such as masticatory cycle, duration and length of lateral excursions, and impaired muscle function which reflect a neuromuscular adaptation to achieve a masticatory cycle with continuous movement and avoiding possible tooth interferences^22,29^. Since this malocclusion rarely self-corrects, the persistence of posterior crossbite may cause alterations in muscle strength during the eruption and establishment of the permanent dentition^11,15,17,26,30^. Moreover, children in the primary dentition with a long-faced tendency were more likely to have crossbite in this study; also, Allen, et al.^1^ (2003) observed that children with longer lower face height and smaller effective maxillary to mandibular skeletal width ratio were more likely to have crossbite, which suggests that craniofacial asymmetries may be a consequence of this malocclusion. Katz, Rosenblatt and Gondim^12^ (2004) did not find significant differences in facial morphology in preschool children with functional crossbite, although direct comparisons are difficult to make, since different results can occur due to variations in ethnicity, age, and method of analysis.

Past studies observed that subjects with strong or thick mandibular elevator muscles have wider transversal head dimensions, and tendencies towards a rectangular shape of the face^13,14,24^. Further, it was shown that masticatory muscles volume exert influence on the size of their adjacent local skeletal sites where the muscles are inserted and on the muscle force is exerted^16^, and a significant correlation between bite force and craniofacial morphology may be observed in preadolescents^9^. This study did not find significant correlation between facial morphology and the magnitude of bite force, which could be attributed to the differences in sample size, methodology, and sample age on comparing the mentioned studies, since this relationship may be less apparent in younger children. Gender differences for facial morphology and bite force were not considered, since they become significant at older ages^11,21,26^. Only in PC group, weight and height were significantly correlated with bite force; the influence of body variables on the magnitude of bite force seems to be controversial in the literature, mainly in young subjects. Rentes, Gavião and Amaral^25^ (2002) found only 6 and 5% variability in maximum bite force could be explained by weight and height, respectively.

## CONCLUSIONS

In the studied sample, it was observed that sucking habits played an important role in the etiology of crossbite, and such condition was related with a decrease in bite force magnitude and longface tendency. Impaired masticatory muscles function and compromised facial esthetics may be consequences of an untreated posterior crossbite with functional shifts. Therefore, such alterations related to this malocclusion may be a reason for early intervention and elimination of factors inhibiting dental arch development, thus providing skeletal correction while the child is still growing^1,28^. But controversy still exists in the literature as to the most appropriate time to treat this condition, and future studies are needed to assess long-term outcomes and analyze costs and possible side effects of the early interventions.
